# Prevalence and factors associated with hypertension among school children and adolescents in urban and semi‐urban areas in Cameroon

**DOI:** 10.1111/jch.14309

**Published:** 2021-06-21

**Authors:** Chris Nadège Nganou‐Gnindjio, Doris Bibi Essama, Jan René Nkeck, Patrick Yvan Tchebegna, Kiliane Maeva Tchatchouang, Aurel Tankeu, Joseph Kamgno

**Affiliations:** ^1^ Faculty of Medicine and Biomedical Sciences University of Yaoundé 1 Yaoundé Cameroon; ^2^ Cardiology Department Yaoundé Central Hospital Yaoundé Cameroon; ^3^ Université Des Montages Bangangte Cameroon

**Keywords:** adolescents, Cameroon, children, hypertension, semi‐urban, urban

## Abstract

Few data to date exist on pediatric hypertension (PH) prevalence and risk factors in semi‐urban areas in Cameroon, and they are believed to be the same as urban areas. These data are needed to design targeted preventive strategies and contribute to reducing the burden of PH in Cameroon and countries with equivalent standards of care. The authors conducted a cross‐sectional study, from November, 2017 to June, 2018, in primary and secondary schools, from semi‐urban (Bamboutos, West Region) and urban (Mfoundi, Center Region) settings in Cameroon, including children and adolescent aged between 3 and 19 years, recruited on a stratified probability sampling. PH was defined according to the American Academy of Pediatrics 2017. Overall, 1001 and 842 participants were, respectively, included in urban and semi‐urban areas. The overall average age was 13.9 ± 4.03 years, and two‐thirds were girls. Overweight and obesity were more prevalent in urban area (overweight: 17.1%; obesity: 5.9%), compared to semi‐urban (overweight: 1.1% and obesity: 0.8%) (*p* < .001). The prevalence of hypertension was higher in urban (12%) than semi‐urban areas (8.6%) (*p* = .01). We have identified as factors associated with PH: age > 14 years (OR = 3.18 [1.6; 6.2]) and secondary level of education (OR = 2.5 [1.2; 5.5]) in urban areas; family history of hypertension (OR 1.93 [1.1; 3.4] in semi‐urban areas. PH prevalence is higher in urban than semi‐urban areas, and the associated factors are not the same. Policies to address hypertension in the pediatric population must be targeted and tailored to the different population profiles.

## 1 INTRODUCTION

There is a progressive increase in the prevalence of hypertension among children and adolescents living in sub‐Saharan Africa (SSA). The currently available epidemiological data indicate prevalences ranging from 0.3 to 24.08%.^[^ ^1^ ^]^ Several facts should alert public health policies in the fight against this scourge. Pediatric hypertension (PH) is unfortunately still underdiagnosed and remains for a long time unrecognized until the occurrence of complications.^[^ ^2^, ^3^ ^]^ The prevalence of major modifiable risk factors such as overweight and obesity is constantly increasing in children and adolescents living in Africa.^[^ ^4^ ^]^ It calls us for regular and broader screening for hypertension in pediatric populations and rigorously addresses its potential risk factors. Therefore, it is important to routinely update epidemiological data on PH in SSA to improve the public health strategies implemented at the local, regional, and national levels.

Policies to address hypertension in the pediatric population must be targeted and tailored to the different population profiles identified beforehand.^[^ ^5^ ^]^ It has been shown that environmental factors influence the prevalence of obesity and overweight, which are major risk factors for PH.^[^ ^4^ ^]^ Indeed, obesity‐promoting factors like sedentary lifestyle and obesogenic diet are related to the standard of living and the area of residence, whether it is urban, rural, or semi‐urban in Cameroon.^[^ ^6^, ^7^ ^]^ Although their significance in shaping preventives strategies, few data to date exist on PH's prevalence and risk factors in semi‐urban areas in Cameroon and are believed to be the same as urban areas. The present study was conducted in Cameroon, a microcosm in SSA and will provide more information on this topic to improve preventive medicine strategies to reduce the burden of PH in Cameroon and countries with equivalent standards of care.

## 2 METHODS

### 2.1 Study design and settings

We conducted a cross‐sectional study, during 8‐month from November 1, 2017 to June 30, 2018, in primary and secondary schools, from urban and semi‐urban settings in Cameroon, defined according to the criteria of the “*Institut National de la Statistique et des Etudes Economiques* (INSEE, 1992)”.^[^ ^8^ ^]^

The urban setting was the Department of Mfoundi, whose capital is Yaoundé, Cameroon's capital. It is located in the Center region and is the most developed department in the country, covering 297 km², comprising seven administrative divisions with a population of 3 525 036 inhabitants (2010), representing 11 869 inhabitants/km^2^. It has 36 schools (primary and secondary).^[^ ^9^ ^]^

The semi‐urban setting was the Bamboutos department, with the city of Mbouda as its capital. It is located in the West region of Cameroon, a predominantly semi‐urban region. It comprises four districts, with a surface area of 437 km² and a population of approximately 175 986 inhabitants (2010), representing 402 inhabitants/km². It has 40 schools (primary and secondary).^[^ ^9^ ^]^

### 2.2 Participants

We have included all children and adolescent, aged between 3 and 19 years old (Adolescents between 10 and 19 years old) attending public and private elementary schools, high schools, and colleges located in the study sites, having consented to participate in the study and whose parents or legal guardians have given their written and informed assent. Participants who were registered but absent on the day of data collection were excluded, as were all participants already known to be hypertensive or taking antihypertensive medication.

### 2.3 Sample size estimation

The minimum sample size was estimated at 827 participants for each group, using the formula for comparison of proportions between two groups contained in the article by Bouyer and Colleagues.^[^ ^10^ ^]^ For this purpose, we defined the statistical risk at 5%, the power at 80%. We used the urban prevalence (2.2%) of hypertension in primary and secondary schools in the city of Yaoundé obtained in 2014 by Donmani.^[^ ^11^ ^]^ In the absence of a study, we used an estimated value of 1% for rural prevalence.

#### 2.3.1 Sampling

We used stratified probability sampling with two levels of stratification.

**First level: choice of schools**

It was done according to the average annual cost of school fees (exchange rates 1 USD = 538 CFA francs). The schools in each department of the different urban and semi‐urban areas were classified into three strata: (1) The stratum of low school fees (< 100 000 CFA francs) subdivided into very low (< 60 000 CFA francs, and moderately low (between 60 001 and 1 00 000 CFA francs); (2) stratum of medium school fees (between 1 00 001 and 1 50 000 CFA francs); and (3) stratum of high school fees (> 1 50 000 CFA francs). We systematically drew two schools (one primary and one secondary) from each stratum without discount using Research Randomizer version 4.0. Sixteen schools (8 primary and 8 secondaries) were selected in both urban and semi‐urban areas. Subsequently, we applied to the administrations of each school for permission to study and obtained approval for 23 of the 32 schools selected.

**Second level: selection of classrooms**

We formed three strata within the nursery, primary, and secondary schools in each setting. All schools were classified according to the French education system. For nursery and primary schools, the first stratum went from the *petite section* to *SIL*; the second stratum went from the *cours préparatoire* to *the cours élémentaire II*; and the third stratum included the *cours moyen I* and *cours moyen II*. For secondary schools: the first stratum was from the class of *sixième* to the class of *quatrième*; the second stratum included the class of *troisième* and *seconde*, and the third stratum included the class of *première* and *terminale*. Using Research Randomizer version 4.0, we systematically drew one classroom for each stratum and each school selected above. We had administrative authorization, making a total of 138 primary and secondary classes.

### 2.4 Data collection

After obtaining ethical clearance from the Institutional Ethics Committee of the *Université des Montages*, and administrative authorizations from the various study sites, we recruited all eligible students. Each participant and his/her parent/guardian were informed of the avoidance (over the last 48 h) of consuming products containing caffeine, tobacco, alcohol, and any medication that could influence blood pressure (BP). Each participant with a parent/guardian was administered a pretested questionnaire under the supervision of trained investigators. The questionnaire reported participants' age, sex, a family history of diabetes, hypertension, and obesity on the 1^st^ degree, type of breastfeeding from birth to 6 months. Subsequently, we proceeded to take anthropometric parameters, including weight estimated to the nearest 0.1 kg using a CAMRY ® brand scale, height measured using a stadiometer to the nearest 0.1 cm, allowing calculation of the body mass index. According to CDC (Center for Disease Control and Prevention) criteria, overweight was defined for a BMI between the 85th and 95th percentiles, obesity for a BMI above the 95th percentile for age, BMI was normal between the 5th and < 85th percentile, and underweight < 5th percentile.^[^ ^12^ ^]^ Excess weight includes obese and/or overweight patients.

### 2.5 Blood pressure measurement and hypertension definition

Blood pressure was measured with an OMRON ® electronic BP monitor with an adapted cuff after a 15‐min rest period. We performed three measurements spaced 10 min apart, and BP was defined as the average of the last two measurements. Hypertension was defined in children and adolescents based on the recommendations of the American Academy of Pediatrics 2017^[^ ^13^ ^]^: normal BP: BP < 90th percentile for age, sex, and height (< 13 years); or < 120/< 80 mm Hg (for adolescents ≥13 years); high BP: BP ≥ 90th percentile and < 95th percentile for age, sex, and height (< 13 years); or 120–129 systolic and diastolic < 80 mm Hg for adolescents ≥13 years; hypertension: ≥95th percentile for age, sex, and height (< 13 years); or ≥ 130/80 mm Hg in adolescents ≥13 years; grade I hypertension: BP ≥95th percentile and < 95th percentile + 12 mm Hg for age, sex, and height (< 13 years); or 130–139 systolic and/or 80–89 mm Hg diastolic in adolescents ≥ 13 years; grade II hypertension: BP ≥95th percentile + 12 mm Hg for age, sex, and height (< 13 years); or > 140/90 mm Hg for adolescents ≥13 years. Systolic and diastolic hypertension were defined for the previously specified age‐specific systole and diastole values, respectively. Pre‐hypertension has been defined according to the fourth report on the diagnosis, evaluation, and treatment of high BP in children and adolescents. We consider a BP above the 90th and less than the 95th percentile for age, height, and weight before 12 years, and ≥120/80 but < 95th percentile after 12 years old.^[^ ^14^ ^]^ Hypertension and prehypertension in patients 18 and 19 years of age were defined according to the Eighth Joint National Committee (JNC8).^[^ ^15^ ^]^ Participants who were diagnosed with a PH were referred for appropriate management.

### 2.6 Statistical analysis

Data were analyzed using SPSS version 20.0 software. Their mean and standard deviation presents continuous variables. Categorical variables are presented with their effective and proportions. The Student's t‐test was used to compare means after checking for normal distribution of the sample. The Chi‐square test was used to compare proportions. After multivariate analysis, the associated factors were determined with logistic regression and presented with their adjusted odds ratio (OR) and 95% confidence interval. For all the tests used, the significance level was set at 0.05.

## 3 RESULTS

Two thousand five hundred students were identified, but 1843 were finally included, comprising 1001 in urban areas and 842 in semi‐urban areas. Six hundred fifty‐seven students could not be recruited because they did not consent to participate in the study or were absent on the day of data collection. Students from public schools were more represented (urban area (524; 54.8%), semi‐urban area (358; 57.8%)), as well as secondary school students (urban area (816; 81.5%), semi‐urban area (736; 87.4%)).

### 3.1 Characteristics of the sample

The overall average age was 13.9 (4.03) years, and the most represented age group in both urban and semi‐urban areas was [15; 19 years] (57.3%). The average age of participants in semi‐urban areas was slightly higher than those in urban areas (15 ±3.7 vs. 13.4 ±4.7, *p* < 0.001). Girls were in the majority, representing two‐thirds of the sample in both urban and semi‐urban areas. Overweight and obesity were more prevalent in urban area (overweight: 17.1%; obesity: 5.9%) compared to semi‐urban (overweight: 1.1% and obesity: 0.8%) (*p* < .001). Mixed breastfeeding in the first six months of life was most used in the urban area (17%) than semi‐urban (13.1%). **Table** **1** summarizes the characteristics of the different groups.

**TABLE 1:** Characteristics of the study population

Variables	Overall	Urban area	Semi urban area	*p* value
N	1843	1001	842	
Female, *n* (%)	1162 (63)	658 (65.7)	504 (59.9)	**.009**
Mean age, year, (SD)	14.1 (4.15)	13.4 (4.7)	15 (3.7)	<.001^*^
Age range, *n* (%)				
[3; 6[	158 (8.6)	107 (10.7)	51 (6.1)	**<.001**
[6; 9[	104 (5.6)	67 (6.7)	37 (4.4)	
[9; 12[	189 (10.3)	152 (15.2)	37 (4.4)	
[12; 15[	336 (18.2)	202 (20.2)	134 (15.9)	
[15; 19]	1056 (57.3)	473 (47.3)	583 (69.2)	
School, *n* (%)				
Public	882 (56)	524 (54.8)	358 (57.8)	.3
Private secular	485 (30.8)	308 (32.2)	177 (28.6)	
Private religious	209 (13.3)	125 (13.1)	84 (13.6)	
Level of education, *n* (%)				
Nursery school	135 (7.3)	104 (10.4)	31 (3.7)	**<.001**
Primary school	156 (8.5)	81 (8.1)	75 (8.9)	
Secondary school	1552 (84.2)	816 (81.5)	736 (87.4)	
Family history of diabetes, *n* (%)	405 (22)	214 (21.4)	191 (22.7)	.5
Family history of hypertension, (%)	509 (27.6)	286 (28.6)	223 (26.5)	.3
Family history of obesity, *n* (%)	433 (23.5)	250 (25)	183 (21.7)	.1
Type of feeding (first 6 months), n (%)				
Exclusive breastfeeding	1481 (80.4)	781 (78)	700 (83.1)	**.02**
Exclusive artificial feeding	82 (4.4)	50 (5)	32 (3.8)	
Mixed breastfeeding	280 (15.2)	170 (17)	110 (13.1)	
BMI classification, n (%)				
Underweight	56 (3)	48 (4.8)	8 (1)	**<.001**
Normal	1541 (83.6)	723 (72.2)	818 (97.1)	
Overweight	180 (9.8)	171 (17.1)	9 (1.1)	
Obese	66 (3.6)	59 (5.9)	7 (0.8)	

BMI: body mass index.

The *p*‐values were obtained after comparing the proportions of the crossed variables by the chi‐square test of independence, except for ^*^ obtained after comparing means between urban and semi‐urban participants using the Student T‐test.

### 3.2 Prevalence of pediatric hypertension in urban and semi‐urban settings

The prevalence of hypertension in the whole sample was 10.4%. It was higher in urban areas (120; 12%) compared with semi‐urban areas (72; 8.6%) (*p* = .01). Hypertension was predominantly graded at grade 1 (133/192). 10% of the children had prehypertension. The prevalence of hypertension was more elevated in females incoming from urban areas (65.8%) than in semi‐urban areas (54.2%). There was no difference in hypertension prevalence with age groups both in urban and semi‐urban areas. More overweight and obese participants with hypertension were in urban than semi‐urban areas (*p* < .001). **Table** **2** summarizes the different prevalence of hypertension in the sample.

**TABLE 2:** Prevalences of hypertension in children and adolescents originated from urban and semi‐urban area in Cameroon

Variables	Overall	Urban area	Semi‐urban area	*p*‐value*
N (%)	1843 (100)	1001 (100)	842 (100)	
Pre‐hypertension	185 (10)	89 (8.9)	96 (11.4)	.07
Hypertension	192 (10.4)	120 (12)	72 (8.6)	**.01**
Grade 1 hypertension	133 (7.2)	84 (8.4)	49 (5.8)	**.03**
Grade 2 hypertension	59 (3.2)	37 (3.7)	22 (2.6)	.19
Systolic hypertension	96 (5.2)	69 (6)	37 (4.6)	**.02**
Diastolic hypertension	133 (7.2)	89 (8.9)	44 (5.2)	**<.01**
Hypertension in Males	74 (38.5)	41 (34.2)	33 (45.8)	.35
Hypertension in Females	118 (61.5)	79 (65.8)	39 (54.2)	**.01**
Hypertension by age groups				
[3; 6[	18 (9.4)	13 (10.8)	5 (6.9)	.2
[6; 9[	10 (5.2)	7 (5.8)	3 (4.2)	
[9; 12[	9 (4.7)	8 (6.7)	1 (1.4)	
[12; 15[	24 (12.5)	16 (13.3)	8 (11.1)	
[15; 19]	131 (68.2)	76 (63.3)	55 (76.4)	
Hypertension by BMI groups				
Normal	147 (76.6)	76 (63.3)	71 (98.6)	**<.001**
Overweight	28 (14.6)	27 (22.5)	1 (1.4)	
Obese	11 (5.7)	11 (9.2)	0 (0)	

BP: blood pressure; BMI: body mass index.

*p‐values were obtained after comparing the proportions of the crossed variables by the chi‐square test of independence.

### 3.3 Factors associated with pediatric hypertension in urban and semi‐urban settings

In the urban setting, the factors associated with hypertension were: age > 14 years (OR = 3.18 [1.6; 6.2]) and secondary level of education (OR = 2.5 [1.2; 5.5]). In the semi‐urban setting, only the family history of hypertension (OR 1.93 [1.1; 3.4] was found as an associated factor. There was no association with excess weight (obesity and overweight) in both areas (**Table** **3**).

**TABLE 3:** Factors associated with hypertension among children and adolescent in urban and semi‐urban areas in Cameroon

Variables	Urban area *N* = 1001 (100%)				Semi‐urban area *N* = 842 (100%)			
	**Hypertension**		**OR [95% CI]**	***p*‐value**	**Hypertension**		**OR [95% CI]**	***p*‐value**
	**Yes** 120 (12)	**No** 881 (98)			**Yes** 72 (8.6)	**No** 770 (91.4)		
Sex								
Female	79 (65.8)	579 (65.7)	1	.5	39 (54.2)	465 (60.4)	1	.25
Male	41 (34.2)	302 (34.3)	1.15 [0.7; 1.7]		33 (45.8)	305 (39.6)	1.32 [0.8; 2.2]	
Age								
< 14	32 (26.7)	384 (43.6)	1	**.001**	12 (16.7)	176 (22.9)	1	.4
≥14	88 (73.3)	497 (56.4)	**3.18 [1.6; 6.2]**		60 (83.3)	584 (77.1)	1.5 [0.5; 3.9]	
Level of education								
Primary or nursery	21 (17.5)	164 (18.6)	1	**.01**	7 (9.7)	99 (12.9)	1	.87
Secondary	99 (82.5)	717 (81.4)	**2.5 [1.2; 5.5]**		65 (90.3)	671 (87.1)	1.1 [0.3; 3.7]	
Family history of diabetes								
No	25 (20.8)	189 (21.5)	1	.9	17 (23.6)	174 (22.6)	1	.88
Yes	95 (79.2)	692 (78.5)	1 [0.6; 1.7]		55 (76.4)	596 (77.4)	0.9 [0.5; 1.8]	
Family history of hypertension								
No	39 (32.5)	247 (28)	1	.2	26 (36.1)	197 (25.6)	1	**.02**
Yes	81 (67.5)	634 (72)	0.7 [0.5;1.2]		46 (63.9)	573 (74.4)	**1.93 [1.1; 3.4]**	
Family history of obesity								
No	33 (27.5)	217 (24.6)	1	.4	14 (19.4)	169 (21.9)	1	.35
Yes	87 (72.5)	664 (75.4)	0.8 [0.5; 1.34]		58 (80.6)	601 (78.1)	0.7 [0.4; 1.4]	
Type of feeding (0 to 6 months)								
Exclusive breastfeeding	102 (85)	679 (77.1)	1	.09	60 (83.3)	640 (83.1)	1	.93
Exclusive artificial feeding or mixed	18 (15)	202 (22.9)	0.8 [0.5; 1.3]		12 (16.7)	130 (16.9)	1 [0.5; 2]	
Excess weight			0.16					
No	82 (68.3)	689 (78.2)	1		71 (98.6)	755 (98.1)	1	.75
Yes	38 (31.7)	192 (21.8)	1.38 [0.8; 2.2]		1 (1.4)	15 (1.9)	1.39 [0.2; 10.9]	

Excess weight = BMI≥85th percentile.

## 4 DISCUSSION

Data on PH in Cameroon and SSA are needed and must be updated to guide public health strategies. It is also important to determine the epidemiological profile of target populations to conduct efficient interventions. In Cameroon, few studies have addressed hypertension in children and adolescents by focusing on geographic origin. In the present study, we found a higher prevalence in urban areas (12%) than semi‐urban areas (8.6%), and the associated factors were not the same in the two regions.

Pediatric hypertension has an ever‐increasing prevalence in SSA, where it remains nevertheless under‐diagnosed. Several African authors who have worked on the patients have found variable prevalence depending on the population, 13.7% in South Africa by Bhimma and Colleagues, 13.6% in boys and 16.5% in girls by Nkeh‐Chungag and Colleagues., also in South Africa, 5.4% in Ghana by Amponsem‐Boateng and Colleagues, 9% in Nigeria by Amadi and Colleagues, and Ezeudu and Colleagues also in Nigeria (6.3%) ^[^ ^16^, ^17^, ^18^, ^19^, ^20^ ^]^. These prevalences were synthesized by Noubiap and Colleagues in a systematic review in SSA, revealing an overall prevalence of 5.5%, and Song and Colleagues who found a prevalence of 4% in a global systematic review.^[^ ^21^, ^22^ ^]^ In Cameroon, few population data are available, the most recent being Chelo and Colleagues survey (1.6%), which found a lower prevalence than ours. Still, they have worked on a population of 5–17 years old, with younger average age (9 ± 5 years), all in elementary school.^[^ ^23^ ^]^ Hypertension in children and adolescents is, therefore, a public health problem that should be detected and treated well before it occurs, particularly in patients with prehypertension who also represent a significant proportion, 10% in our study, 2.5% in the survey by Okpokowuruk and Colleagues in a semi‐urban environment, 8% in the survey by Amponsem‐Boateng and Colleagues, and 26.7% in the study by Bhimma and Colleagues.^[^ ^16^, ^18^, ^24^ ^]^

In urban and semi‐urban areas, the characteristics of the populations are not always the same, starting with diet and physical activity, which are known to be significant determinants of hypertension and are strongly influenced by geographical origin. Indeed, we found that the prevalence in urban areas was significantly higher than in semi‐urban areas. In the African literature, some authors have studied the patients, notably Obika and Colleagues, who found no difference in the prevalence of hypertension in urban, rural, and semi‐urban areas in Nigeria.^[^ ^25^ ^]^ Agyemang and Colleagues found lower BP figures in rural areas, with no difference between semi‐urban and rural areas.^[^ ^26^ ^]^ Furthermore, we found a high prevalence of systolic and diastolic hypertension in urban areas compared to semi‐urban areas. This contrasts with the Ejike and Colleagues study results, which observed an increase in systolic BP in urban areas compared to non‐urban areas and an increase in diastolic BP in non‐urban areas compared to urban areas.^[^ ^27^ ^]^ However, the profile of our populations was not the same; they worked on a population of adolescents only. This disparity in prevalence relates to the epidemiological gap of the population and calls for a targeted strategy, as pointed out by Noubiap and Colleagues.^[^ ^5^ ^]^

Several factors may influence the occurrence of hypertension in children and adolescents. We found that these factors differed in urban and semi‐urban settings. Indeed, only age and high school education significantly increased the risk of hypertension in urban areas. In semi‐urban areas, only a family history of hypertension was an associated factor. Age, being a known risk factor for PH, reflects the accumulation and progression of different risk factors and is dependent on education level.^[^ ^22^, ^23^ ^]^ Obesity and overweight are known as classical risk factors for hypertension in urban children and adolescents. They have been found by Jobe and Colleagues in urban West Africa, Bhimma and Colleagues, Nkeh‐Chungag and Colleagues in South Africa, Chelo and Colleagues in Cameroon, and Okpokowuruk and Colleagues in Nigeria.^[^ ^16^, ^17^, ^23^, ^24^, ^28^ ^]^ The absence of these risk factors in our population suggests that other associated factors could play a role. Notably, the diet that is principally rich in fruit and vegetables and low in salt has not been evaluated, regular physical activity, birth weight, and birth term, for which data could not be collected.

In the light of these results, it seems crucial to understand that the epidemiological profile of children and adolescents with hypertension is not totally the same in urban and semi‐urban settings. The implications of these results could be at several levels, in particular primary prevention strategies that need to be adjusted at the community, regional, and national levels; the need to regularly update data; and the need to evaluate long‐term follow‐up to control potential risk factors and address them appropriately. The interpretation of the results of our study must nevertheless take into account several limitations: the possibility of a white coat effect and/or masked hypertension, which were not evaluated during our research and whose impact on prevalence is not negligible, especially in SSA^[^ ^29^ ^]^; the different references defining hypertension in children and adolescents, which limits the comparison with certain studies; and the analysis of specific risk factors for hypertension, notably diet, which unfortunately are not yet well codified in our context.

## 5 CONCLUSIONS

PH prevalence is higher in urban than semi‐urban areas in Cameroon, and the associated factors are not the same. Policies to address hypertension in the pediatric population must be targeted and tailored to the different population profiles. However, it is necessary to obtain long‐term follow‐up data to better define the risk factors for PH in our context, both in urban and semi‐urban areas.

## CONFLICT OF INTEREST

The authors declare that they have no competing interests.

## AUTHOR CONTRIBUTIONS

Chris Nadège Nganou‐Gnindjio, Aurel Tankeu, and Joseph Kamgno involved in the conception and design of the research. Chris Nadège Nganou‐Gnindjio, Patrick Yvan Tchebegna, and Kiliane Maeva Tchatchouang involved in the acquisition, analysis, and interpretation of the data. Jan René Nkeck, Doris Bibi Essama, and Patrick Yvan Tchebegna involved in statistical analysis. Joseph Kamgno involved in the supervision of the work. Jan René Nkeck, Doris Bibi Essama, and Patrick Yvan Tchebegna involved in drafting the manuscript. All the authors involved in the critical revision of the manuscript for important intellectual content and approbation of the final version.
